# (*E*)-Dimethyl 2-(6-benzoyl-7-hydr­oxy-4-methoxy­carbonyl-2-oxo-2*H*-chromen-8-yl)but-2-enedioate

**DOI:** 10.1107/S1600536808020990

**Published:** 2008-07-16

**Authors:** Robabeh Baharfar, S. Mohammad Vahdat, S. Meysam Baghbanian

**Affiliations:** a Department of Chemistry, University of Mazandaran, 47415 Babolsar, Iran

## Abstract

The mol­ecule of the title compound, C_24_H_18_O_10_, a previously unknown coumarin derivative, contains methoxy­carbonyl, 2-butenedioate and benzoyl groups aligned at angles of 28.04 (2), 76.89 (3) and 42.48 (13)°, respectively, to the plane of the coumarin ring system. Intra­molecular O—H⋯O hydrogen bonding between hydr­oxy and carbonyl groups and weak inter­molecular C—H⋯O hydrogen bonding is present in the crystal structure. The two carbon atoms and attached H atom of the ethylene bond are disordered over two positions, with site occupancy factors of *ca* 0.9 and 0.1.

## Related literature

For general background, see: Maeda (1984 ▶); Parrish *et al.* (1974 ▶); Troste & Toste (1996 ▶); Khalfan *et al.* (1987 ▶); Hooper *et al.* (1982 ▶); Morris & Russell (1971 ▶).
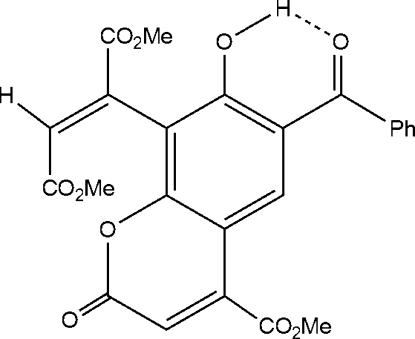


## Experimental

### 

#### Crystal data

C_24_H_18_O_10_
*M* *_r_* = 466.38
Orthorhombic, 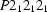

*a* = 9.2661 (17) Å
*b* = 11.508 (2) Å
*c* = 19.661 (4) Å
*V* = 2096.5 (7) Å^3^
*Z* = 4
Mo *K*α radiation
μ = 0.12 mm^−1^
*T* = 120 (2) K
0.3 × 0.2 × 0.2 mm

#### Data collection

Bruker SMART 1000 CCD area-detector diffractometer
Absorption correction: multi-scan (*SADABS*; Sheldrick, 2008 ▶) *T*_min_ = 0.973, *T*_max_ = 0.979
12547 measured reflections
2864 independent reflections
2194 reflections with *I* > 2σ(*I*)
*R*_int_ = 0.046

#### Refinement

*R*[*F* ^2^ > 2σ(*F* ^2^)] = 0.046
*wR*(*F* ^2^) = 0.113
*S* = 1.00
2864 reflections
323 parameters
1 restraint
H atoms treated by a mixture of independent and constrained refinement
Δρ_max_ = 0.35 e Å^−3^
Δρ_min_ = −0.25 e Å^−3^

### 

Data collection: *SMART* (Bruker, 1998 ▶); cell refinement: *SAINT-Plus* (Bruker, 2008 ▶); data reduction: *SAINT-Plus*; program(s) used to solve structure: *SHELXTL* (Sheldrick, 2008 ▶); program(s) used to refine structure: *SHELXTL*; molecular graphics: *SHELXTL*; software used to prepare material for publication: *SHELXTL*.

## Supplementary Material

Crystal structure: contains datablocks I, global. DOI: 10.1107/S1600536808020990/xu2430sup1.cif

Structure factors: contains datablocks I. DOI: 10.1107/S1600536808020990/xu2430Isup2.hkl

Additional supplementary materials:  crystallographic information; 3D view; checkCIF report

## Figures and Tables

**Table 1:** Hydrogen-bond geometry (Å, °)

*D*—H⋯*A*	*D*—H	H⋯*A*	*D*⋯*A*	*D*—H⋯*A*
O3—H3*O*⋯O8	0.99 (6)	1.65 (6)	2.541 (4)	149 (5)
C13—H13*C*⋯O3^i^	0.96	2.64	3.467 (5)	145
C15—H15*C*⋯O2^ii^	0.96	2.53	3.342 (5)	142
C22—H22*A*⋯O8^iii^	0.93	2.49	3.316 (5)	149
C24—H24*A*⋯O5^iv^	0.96	2.67	3.392 (5)	133

Symmetry codes: (i) 
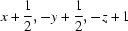
; (ii) 
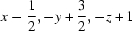
; (iii) 
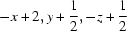
; (iv) 
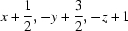
.

**Fig. 1.**
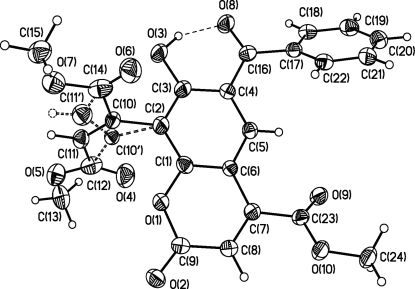
The molecular structure of the title compound with 50% probability displacement ellipsoids for non-H atoms. Dashed line indicates hydrogen bonding and double dashed lines indicate the minor component of the disordered structure.

## supplementary crystallographic information

### Comment

Coumarin derivatives are used as laser dyes (Maeda, 1984). Some of them are found in natural products and exhibit antifungal and anticoagulant properties (Parrish *et al.*,  1974; Troste & Toste, 1996). They have been found to possess a wide variety of uses in the perfumery industry, as avour enhancers, sunscreens, laser dyes (Khalfan *et al.*,  1987) and in the pharmaceutical industry (Hooper *et al.*,  1982; Morris & Russell, 1971). We have recently synthesized a series of 7-hydroxy coumarins based on a direct, efficient and operationally convenient approach. This paper reports the synthesis and structure of the title compound, which is one of the products of this reaction.

In the molecular structure (Fig. 1) of the title compound, atoms C10, C11 and H11A are disordered over two sites with occupancy ratio of 0.85:0.15. The inclinations of the planes of the metoxycarbonyl (defined by atoms O9-C23-O10-C24), hydroxy group (defined by atoms C3-O3-H3O), olefin (defined by atoms C12-C11-C10-C14) and benzoyl substituents (defined by atoms C16 to C22) with respect to the coumarin ring system are 28.04 (2), 12.1 (4), 76.89 (3) and 42.48 (13)°, respectively. Torsion angle between hydroxy group and carbonyl of benzoyl group is 0.7 (5)°. Therefore, these two groups are coplanar and form an intramolecular O-H···O hydrogen bonding. Torsion angle between olefin substituent and coumarin moiety is 106.0 (5)°. E-configuration was assigned to the geometry of olefinic bond on the basis of torsion angle of 176.8 (4)° between two methoxy carbonyl groups. Torsion angle between phenyl and carbonyl of benzoyl group is 140.9 (4)°. The crystal structure contains weak intermolecular C—H···O hydrogen bonding (Table 1).

### Experimental

To a magnetically stirred solution of 2,4-dihydroxy benzophenone (0.43 g, 2 mmol) and triphenylphosphine (0.52 g, 2 mmol) in 10 ml CH_2_Cl_2_ was added dropwise at 263 K over 10 min dimethyl acetylenedicarboxylate (0.45g, 2 mmol). The reaction mixture was then allowed to warm up to room temperature and stand for 48 h. The solvent was removed under reduced pressure and the residue was separated by silica gel column chromatography (Merck 230-400 mesh) using n-hexane–ethyl acetate as eluent. The single crystals of the title compound were obtained form the n-hexane–ethyl acetate solution. Anal. Calcd. for C_24_H_18_O_10_ (466): C, 61.80; H, 3.86 %: Found: C, 61.70; H, 3.77 % .

### Refinement

The ethylene of the dimethyl fumarate moiety is disordered over two sites; occupancies were initially refined and converged to ca 0.867:0.133, and fixed as 0.85:0.15 at final cycles of refinement. The hydroxyl H atom was located in a difference Fourier map and refined isotropically. Other H atoms were placed in calculated positions and refined in riding model with the *U*_iso_(H) = 1.5*U*_eq_(C) (methyl) or 1.2*U*_eq_(C) (others). In absence of significant anomalous scattering effects, Friedel pairs were merged.

### Crystal data

**Table:**

C_24_H_18_O_10_	*D*_x_ = 1.478 Mg m^−^^3^
*M**_r_* = 466.38	Melting point: 457 K
Orthorhombic, *P*2_1_2_1_2_1_	Mo *K*α radiation λ = 0.71073 Å
Hall symbol: P 2ac 2ab	Cell parameters from 557 reflections
*a* = 9.2661 (17) Å	θ = 3–28º
*b* = 11.508 (2) Å	µ = 0.12 mm^−^^1^
*c* = 19.661 (4) Å	*T* = 120 (2) K
*V* = 2096.5 (7) Å^3^	Rhombic, yellow
*Z* = 4	0.3 × 0.2 × 0.2 mm
*F*_000_ = 968	

### Data collection

**Table:**

Bruker SMART 1000 CCD area-detector diffractometer	2864 independent reflections
Radiation source: fine-focus sealed tube	2194 reflections with *I* > 2σ(*I*)
Monochromator: graphite	*R*_int_ = 0.046
*T* = 120(2) K	θ_max_ = 28.0º
φ and ω scans	θ_min_ = 2.1º
Absorption correction: multi-scan(SADABS; Sheldrick, 2008)	*h* = −10→12
*T*_min_ = 0.973, *T*_max_ = 0.979	*k* = −15→13
12547 measured reflections	*l* = −17→25

### Refinement

**Table:**

Refinement on *F*^2^	Secondary atom site location: difference Fourier map
Least-squares matrix: full	Hydrogen site location: inferred from neighbouring sites
*R*[*F*^2^ > 2σ(*F*^2^)] = 0.046	H atoms treated by a mixture of independent and constrained refinement
*wR*(*F*^2^) = 0.113	*w* = 1/[σ^2^(*F*_o_^2^) + (0.0236*P*)^2^ + 2.3937*P*] where *P* = (*F*_o_^2^ + 2*F*_c_^2^)/3
*S* = 1.00	(Δ/σ)_max_ = 0.001
2864 reflections	Δρ_max_ = 0.35 e Å^−^^3^
323 parameters	Δρ_min_ = −0.25 e Å^−^^3^
1 restraint	Extinction correction: none
Primary atom site location: structure-invariant direct methods	

### Special details

**Table:**

Experimental. ^1^H NMR (500 MHz, CDCl_3_): δ = 3.66, 3.82 and 3.86 (9 H, 3 s, 3 OCH_3_), 6.82, 7.27 and 8.85 (3 H, 3 s, 3 CH), 7.54 -7.76 (5 H, m, CH, Aromatic), 12.90 (1 H, s, OH). ^13^C NMR (125.7 MHz, CDCl_3_): δ = 52.06, 53.11 and 53.18 (3 OCH_3_), 108.11 (CH), 112.32, 116.18, 117.22 (3 C), 128.58, 129.50, 132.13 and 132.74 (4 CH), 134.22, 135.26 and 137.06 (3 C), 141.60 (CH), 156.09 and 158.80 (2 C-O), 163.43, 163.87, 164.66 and 165.25 (4 C=O, Ester), 200.7 (C=O, Ketone). IR (KBr) (νmax /cm^-1^): 3320-3550 (OH), 1735-1750 (C=O, Ketone), 1615-1632 (C=O, Ester), 1400-1435 (C=C). MS, (m/z, %): 466 (9) (M+), 105 (25), 44 (98).
Geometry. All esds (except the esd in the dihedral angle between two l.s. planes) are estimated using the full covariance matrix. The cell esds are taken into account individually in the estimation of esds in distances, angles and torsion angles; correlations between esds in cell parameters are only used when they are defined by crystal symmetry. An approximate (isotropic) treatment of cell esds is used for estimating esds involving l.s. planes.
Refinement. Refinement of F^2^ against ALL reflections. The weighted R-factor wR and goodness of fit S are based on F^2^, conventional R-factors R are based on F, with F set to zero for negative F^2^. The threshold expression of F^2^ > 2sigma(F^2^) is used only for calculating R-factors(gt) etc. and is not relevant to the choice of reflections for refinement. R-factors based on F^2^ are statistically about twice as large as those based on F, and R- factors based on ALL data will be even larger.

### Fractional atomic coordinates and isotropic or equivalent isotropic displacement parameters (Å^2^)

**Table:**

	*x*	*y*	*z*	*U*_iso_*/*U*_eq_	Occ. (<1)
O1	0.8901 (3)	0.6309 (2)	0.47921 (12)	0.0296 (5)	
O2	0.9133 (3)	0.7985 (2)	0.53324 (12)	0.0320 (6)	
O3	0.8287 (3)	0.2634 (2)	0.38265 (14)	0.0382 (7)	
O4	1.0075 (3)	0.4046 (3)	0.55108 (15)	0.0515 (8)	
O5	0.8674 (3)	0.3474 (2)	0.63681 (14)	0.0394 (7)	
O6	0.5738 (3)	0.4577 (3)	0.38390 (15)	0.0469 (7)	
O7	0.4741 (3)	0.4059 (2)	0.48358 (14)	0.0395 (7)	
O8	0.9960 (3)	0.2006 (2)	0.28708 (13)	0.0367 (6)	
O9	1.3022 (3)	0.6689 (2)	0.30924 (13)	0.0331 (6)	
O10	1.2205 (3)	0.8493 (2)	0.33093 (13)	0.0314 (6)	
C1	0.9316 (4)	0.5539 (3)	0.42981 (17)	0.0265 (7)	
C2	0.8588 (4)	0.4490 (3)	0.42914 (19)	0.0312 (8)	
C3	0.8983 (4)	0.3662 (3)	0.38097 (19)	0.0294 (8)	
C4	1.0067 (4)	0.3908 (3)	0.33190 (16)	0.0240 (7)	
C5	1.0769 (4)	0.4973 (3)	0.33484 (16)	0.0241 (7)	
H5A	1.1498	0.5131	0.3037	0.029*	
C6	1.0414 (4)	0.5814 (3)	0.38323 (16)	0.0223 (6)	
C7	1.1059 (4)	0.6962 (3)	0.38800 (16)	0.0244 (7)	
C8	1.0624 (4)	0.7706 (3)	0.43690 (17)	0.0256 (7)	
H8A	1.1037	0.8441	0.4389	0.031*	
C9	0.9531 (4)	0.7394 (3)	0.48650 (17)	0.0263 (7)	
C10	0.7329 (4)	0.4258 (3)	0.4747 (2)	0.0246 (8)	0.85
C11	0.7438 (4)	0.3982 (4)	0.5399 (2)	0.0285 (9)	0.85
H11A	0.6602	0.3865	0.5651	0.034*	0.85
C10'	0.817 (3)	0.4049 (19)	0.5058 (11)	0.022 (4)*	0.15
C11'	0.677 (3)	0.387 (2)	0.5078 (13)	0.038 (6)*	0.15
H11B	0.6332	0.3503	0.5446	0.045*	0.15
C12	0.8891 (5)	0.3854 (3)	0.5746 (2)	0.0380 (9)	
C13	0.9957 (4)	0.3285 (4)	0.67707 (19)	0.0407 (10)	
H13A	0.9693	0.2953	0.7201	0.061*	
H13B	1.0439	0.4012	0.6844	0.061*	
H13C	1.0591	0.2763	0.6535	0.061*	
C14	0.5856 (4)	0.4312 (3)	0.4419 (2)	0.0363 (9)	
C15	0.3335 (4)	0.4193 (4)	0.4522 (2)	0.0417 (10)	
H15A	0.2605	0.3907	0.4825	0.063*	
H15B	0.3306	0.3761	0.4105	0.063*	
H15C	0.3164	0.5000	0.4429	0.063*	
C16	1.0426 (4)	0.3018 (3)	0.28092 (18)	0.0273 (7)	
C17	1.1342 (4)	0.3286 (3)	0.22093 (17)	0.0259 (7)	
C18	1.2264 (4)	0.2416 (3)	0.19724 (18)	0.0297 (8)	
H18A	1.2301	0.1703	0.2194	0.036*	
C19	1.3132 (4)	0.2616 (3)	0.14031 (19)	0.0345 (9)	
H19A	1.3768	0.2048	0.1251	0.041*	
C20	1.3031 (4)	0.3672 (4)	0.1070 (2)	0.0372 (9)	
H20A	1.3613	0.3813	0.0694	0.045*	
C21	1.2084 (4)	0.4517 (3)	0.12855 (19)	0.0339 (8)	
H21A	1.2013	0.5214	0.1048	0.041*	
C22	1.1240 (4)	0.4333 (3)	0.18531 (18)	0.0291 (7)	
H22A	1.0603	0.4906	0.1999	0.035*	
C23	1.2213 (4)	0.7331 (3)	0.33863 (17)	0.0266 (7)	
C24	1.3248 (4)	0.8940 (3)	0.2829 (2)	0.0361 (9)	
H24A	1.3133	0.9766	0.2788	0.054*	
H24B	1.3098	0.8582	0.2393	0.054*	
H24C	1.4205	0.8769	0.2986	0.054*	
H3O	0.884 (6)	0.213 (5)	0.352 (3)	0.078 (17)*	

### Atomic displacement parameters (Å^2^)

**Table:**

	*U*^11^	*U*^22^	*U*^33^	*U*^12^	*U*^13^	*U*^23^
O1	0.0358 (13)	0.0261 (12)	0.0269 (12)	−0.0040 (11)	0.0063 (11)	−0.0025 (10)
O2	0.0351 (14)	0.0322 (13)	0.0289 (13)	0.0018 (11)	0.0021 (11)	−0.0061 (11)
O3	0.0440 (16)	0.0275 (14)	0.0432 (16)	−0.0110 (12)	0.0143 (13)	−0.0061 (12)
O4	0.0523 (19)	0.065 (2)	0.0368 (16)	−0.0114 (17)	−0.0002 (15)	0.0119 (15)
O5	0.0301 (14)	0.0448 (16)	0.0433 (16)	0.0018 (12)	−0.0013 (13)	0.0071 (13)
O6	0.0376 (16)	0.0583 (19)	0.0447 (17)	−0.0016 (14)	0.0041 (14)	−0.0037 (15)
O7	0.0371 (15)	0.0433 (15)	0.0380 (15)	0.0026 (13)	−0.0069 (13)	0.0006 (13)
O8	0.0442 (16)	0.0248 (13)	0.0410 (15)	−0.0064 (12)	0.0104 (13)	−0.0035 (12)
O9	0.0299 (13)	0.0286 (13)	0.0408 (14)	−0.0029 (11)	0.0088 (12)	−0.0057 (12)
O10	0.0309 (13)	0.0264 (13)	0.0369 (14)	−0.0033 (11)	0.0065 (11)	0.0015 (11)
C1	0.0321 (18)	0.0248 (16)	0.0224 (16)	0.0022 (14)	−0.0005 (15)	0.0005 (14)
C2	0.038 (2)	0.0260 (17)	0.0302 (18)	−0.0046 (15)	0.0069 (16)	−0.0010 (15)
C3	0.0318 (18)	0.0239 (17)	0.0327 (18)	−0.0042 (14)	0.0020 (16)	0.0000 (15)
C4	0.0245 (16)	0.0249 (16)	0.0226 (16)	0.0005 (13)	−0.0012 (14)	−0.0015 (14)
C5	0.0246 (16)	0.0242 (16)	0.0236 (16)	0.0009 (13)	0.0001 (14)	0.0010 (14)
C6	0.0236 (15)	0.0218 (15)	0.0214 (15)	−0.0005 (13)	−0.0001 (13)	0.0000 (13)
C7	0.0247 (16)	0.0251 (16)	0.0233 (16)	0.0002 (14)	−0.0024 (14)	0.0011 (14)
C8	0.0266 (17)	0.0236 (16)	0.0266 (16)	−0.0006 (13)	−0.0024 (14)	−0.0009 (13)
C9	0.0286 (18)	0.0240 (16)	0.0263 (17)	0.0009 (14)	−0.0032 (14)	−0.0028 (15)
C10	0.0219 (19)	0.026 (2)	0.026 (2)	−0.0021 (16)	0.0013 (17)	0.0009 (17)
C11	0.025 (2)	0.029 (2)	0.031 (2)	0.0008 (17)	0.0036 (19)	0.0006 (18)
C12	0.043 (2)	0.0306 (19)	0.040 (2)	−0.0043 (18)	−0.0105 (19)	0.0019 (17)
C13	0.036 (2)	0.056 (3)	0.0305 (19)	0.0127 (19)	−0.0005 (17)	0.0111 (19)
C14	0.0253 (18)	0.032 (2)	0.052 (3)	0.0042 (16)	−0.0086 (18)	−0.0091 (18)
C15	0.0271 (19)	0.044 (2)	0.054 (3)	−0.0002 (17)	0.0013 (18)	−0.010 (2)
C16	0.0271 (17)	0.0235 (16)	0.0312 (18)	−0.0002 (14)	−0.0027 (15)	−0.0012 (15)
C17	0.0247 (16)	0.0266 (17)	0.0266 (17)	−0.0026 (14)	−0.0010 (14)	−0.0038 (14)
C18	0.0290 (18)	0.0263 (18)	0.0337 (19)	0.0003 (14)	−0.0036 (15)	−0.0058 (15)
C19	0.0294 (19)	0.036 (2)	0.038 (2)	0.0023 (16)	0.0009 (17)	−0.0102 (17)
C20	0.036 (2)	0.042 (2)	0.033 (2)	−0.0043 (17)	0.0085 (17)	−0.0090 (17)
C21	0.041 (2)	0.0302 (18)	0.0305 (18)	−0.0032 (16)	0.0022 (17)	−0.0036 (16)
C22	0.0325 (18)	0.0254 (17)	0.0294 (17)	0.0007 (15)	−0.0023 (16)	−0.0040 (15)
C23	0.0269 (17)	0.0260 (17)	0.0269 (17)	−0.0043 (14)	−0.0032 (15)	−0.0011 (15)
C24	0.036 (2)	0.0304 (19)	0.042 (2)	−0.0081 (17)	0.0083 (17)	0.0046 (17)

### Geometric parameters (Å, °)

**Table:**

O1—C1	1.370 (4)	C10—C11	1.326 (6)
O1—C9	1.386 (4)	C10—C14	1.511 (5)
O2—C9	1.201 (4)	C11—C12	1.516 (6)
O3—C3	1.347 (4)	C11—H11A	0.9300
O3—H3O	0.99 (5)	C10'—C11'	1.31 (4)
O4—C12	1.211 (5)	C10'—C12	1.52 (2)
O5—C12	1.314 (5)	C11'—C14	1.63 (2)
O5—C13	1.444 (4)	C11'—H11B	0.9300
O6—C14	1.185 (5)	C13—H13A	0.9600
O7—C14	1.351 (5)	C13—H13B	0.9600
O7—C15	1.449 (5)	C13—H13C	0.9600
O8—C16	1.248 (4)	C15—H15A	0.9600
O9—C23	1.200 (4)	C15—H15B	0.9600
O10—C23	1.345 (4)	C15—H15C	0.9600
O10—C24	1.446 (4)	C16—C17	1.485 (5)
C1—C2	1.382 (5)	C17—C18	1.396 (5)
C1—C6	1.405 (5)	C17—C22	1.397 (5)
C2—C3	1.393 (5)	C18—C19	1.397 (5)
C2—C10	1.495 (5)	C18—H18A	0.9300
C2—C10'	1.64 (2)	C19—C20	1.383 (6)
C3—C4	1.421 (5)	C19—H19A	0.9300
C4—C5	1.389 (4)	C20—C21	1.377 (5)
C4—C16	1.471 (5)	C20—H20A	0.9300
C5—C6	1.396 (4)	C21—C22	1.379 (5)
C5—H5A	0.9300	C21—H21A	0.9300
C6—C7	1.454 (4)	C22—H22A	0.9300
C7—C8	1.349 (5)	C24—H24A	0.9600
C7—C23	1.505 (5)	C24—H24B	0.9600
C8—C9	1.451 (5)	C24—H24C	0.9600
C8—H8A	0.9300		
			
C1—O1—C9	122.6 (3)	O4—C12—C10'	91.8 (9)
C3—O3—H3O	104 (3)	O5—C12—C10'	143.9 (10)
C12—O5—C13	115.7 (3)	O5—C13—H13A	109.5
C14—O7—C15	114.0 (3)	O5—C13—H13B	109.5
C23—O10—C24	115.1 (3)	H13A—C13—H13B	109.5
O1—C1—C2	115.8 (3)	O5—C13—H13C	109.5
O1—C1—C6	121.3 (3)	H13A—C13—H13C	109.5
C2—C1—C6	122.9 (3)	H13B—C13—H13C	109.5
C1—C2—C3	118.4 (3)	O6—C14—O7	124.7 (4)
C1—C2—C10	122.1 (3)	O6—C14—C10	120.3 (4)
C3—C2—C10	119.3 (3)	O7—C14—C10	115.0 (3)
C1—C2—C10'	112.2 (8)	O6—C14—C11'	153.2 (11)
C3—C2—C10'	118.4 (8)	O7—C14—C11'	81.3 (11)
O3—C3—C2	117.3 (3)	O7—C15—H15A	109.5
O3—C3—C4	122.0 (3)	O7—C15—H15B	109.5
C2—C3—C4	120.7 (3)	H15A—C15—H15B	109.5
C5—C4—C3	118.6 (3)	O7—C15—H15C	109.5
C5—C4—C16	122.4 (3)	H15A—C15—H15C	109.5
C3—C4—C16	118.9 (3)	H15B—C15—H15C	109.5
C4—C5—C6	121.9 (3)	O8—C16—C4	120.3 (3)
C4—C5—H5A	119.0	O8—C16—C17	117.9 (3)
C6—C5—H5A	119.0	C4—C16—C17	121.7 (3)
C5—C6—C1	117.3 (3)	C18—C17—C22	119.5 (3)
C5—C6—C7	125.2 (3)	C18—C17—C16	117.8 (3)
C1—C6—C7	117.4 (3)	C22—C17—C16	122.6 (3)
C8—C7—C6	120.0 (3)	C17—C18—C19	120.1 (3)
C8—C7—C23	119.6 (3)	C17—C18—H18A	119.9
C6—C7—C23	120.5 (3)	C19—C18—H18A	119.9
C7—C8—C9	122.1 (3)	C20—C19—C18	119.0 (3)
C7—C8—H8A	118.9	C20—C19—H19A	120.5
C9—C8—H8A	118.9	C18—C19—H19A	120.5
O2—C9—O1	117.4 (3)	C21—C20—C19	121.2 (4)
O2—C9—C8	126.0 (3)	C21—C20—H20A	119.4
O1—C9—C8	116.6 (3)	C19—C20—H20A	119.4
C11—C10—C2	124.3 (4)	C20—C21—C22	120.1 (4)
C11—C10—C14	119.5 (4)	C20—C21—H21A	119.9
C2—C10—C14	116.2 (3)	C22—C21—H21A	119.9
C10—C11—C12	121.7 (4)	C21—C22—C17	120.0 (3)
C10—C11—H11A	119.1	C21—C22—H22A	120.0
C12—C11—H11A	119.1	C17—C22—H22A	120.0
C11'—C10'—C12	112 (2)	O9—C23—O10	124.1 (3)
C11'—C10'—C2	108.1 (19)	O9—C23—C7	125.5 (3)
C12—C10'—C2	139.4 (16)	O10—C23—C7	110.4 (3)
C10'—C11'—C14	116 (2)	O10—C24—H24A	109.5
C10'—C11'—H11B	122.0	O10—C24—H24B	109.5
C14—C11'—H11B	122.0	H24A—C24—H24B	109.5
O4—C12—O5	123.7 (4)	O10—C24—H24C	109.5
O4—C12—C11	128.0 (4)	H24A—C24—H24C	109.5
O5—C12—C11	108.4 (4)	H24B—C24—H24C	109.5
			
C9—O1—C1—C2	179.8 (3)	C10—C2—C10'—C12	−165 (3)
C9—O1—C1—C6	−0.2 (5)	C12—C10'—C11'—C14	167.5 (15)
O1—C1—C2—C3	−178.7 (3)	C2—C10'—C11'—C14	−10 (3)
C6—C1—C2—C3	1.3 (5)	C13—O5—C12—O4	−0.4 (6)
O1—C1—C2—C10	6.0 (5)	C13—O5—C12—C11	179.1 (3)
C6—C1—C2—C10	−174.0 (3)	C13—O5—C12—C10'	167.0 (15)
O1—C1—C2—C10'	−35.1 (10)	C10—C11—C12—O4	5.3 (7)
C6—C1—C2—C10'	144.9 (9)	C10—C11—C12—O5	−174.2 (4)
C1—C2—C3—O3	178.1 (3)	C10—C11—C12—C10'	−6.1 (14)
C10—C2—C3—O3	−6.5 (5)	C11'—C10'—C12—O4	177 (2)
C10'—C2—C3—O3	36.8 (10)	C2—C10'—C12—O4	−7(2)
C1—C2—C3—C4	−2.6 (5)	C11'—C10'—C12—O5	8(3)
C10—C2—C3—C4	172.9 (3)	C2—C10'—C12—O5	−176.5 (11)
C10'—C2—C3—C4	−143.9 (10)	C11'—C10'—C12—C11	−11.8 (15)
O3—C3—C4—C5	−177.9 (3)	C2—C10'—C12—C11	164 (3)
C2—C3—C4—C5	2.8 (5)	C15—O7—C14—O6	−2.9 (6)
O3—C3—C4—C16	0.7 (5)	C15—O7—C14—C10	176.0 (3)
C2—C3—C4—C16	−178.6 (3)	C15—O7—C14—C11'	−175.6 (11)
C3—C4—C5—C6	−1.8 (5)	C11—C10—C14—O6	178.6 (4)
C16—C4—C5—C6	179.7 (3)	C2—C10—C14—O6	−3.2 (6)
C4—C5—C6—C1	0.5 (5)	C11—C10—C14—O7	−0.3 (5)
C4—C5—C6—C7	−177.6 (3)	C2—C10—C14—O7	177.8 (3)
O1—C1—C6—C5	179.7 (3)	C11—C10—C14—C11'	−15.0 (18)
C2—C1—C6—C5	−0.3 (5)	C2—C10—C14—C11'	163.2 (18)
O1—C1—C6—C7	−2.0 (5)	C10'—C11'—C14—O6	24 (4)
C2—C1—C6—C7	178.0 (3)	C10'—C11'—C14—O7	−170 (2)
C5—C6—C7—C8	179.7 (3)	C10'—C11'—C14—C10	−3.2 (12)
C1—C6—C7—C8	1.6 (5)	C5—C4—C16—O8	167.3 (3)
C5—C6—C7—C23	−0.2 (5)	C3—C4—C16—O8	−11.2 (5)
C1—C6—C7—C23	−178.3 (3)	C5—C4—C16—C17	−12.8 (5)
C6—C7—C8—C9	0.9 (5)	C3—C4—C16—C17	168.6 (3)
C23—C7—C8—C9	−179.1 (3)	O8—C16—C17—C18	−34.7 (5)
C1—O1—C9—O2	−176.9 (3)	C4—C16—C17—C18	145.5 (3)
C1—O1—C9—C8	2.7 (5)	O8—C16—C17—C22	140.9 (4)
C7—C8—C9—O2	176.5 (3)	C4—C16—C17—C22	−38.9 (5)
C7—C8—C9—O1	−3.1 (5)	C22—C17—C18—C19	3.2 (5)
C1—C2—C10—C11	−78.7 (5)	C16—C17—C18—C19	179.0 (3)
C3—C2—C10—C11	106.0 (5)	C17—C18—C19—C20	−1.8 (5)
C10'—C2—C10—C11	6.5 (13)	C18—C19—C20—C21	−0.7 (6)
C1—C2—C10—C14	103.2 (4)	C19—C20—C21—C22	1.7 (6)
C3—C2—C10—C14	−72.0 (5)	C20—C21—C22—C17	−0.2 (5)
C10'—C2—C10—C14	−171.6 (13)	C18—C17—C22—C21	−2.2 (5)
C2—C10—C11—C12	−1.3 (6)	C16—C17—C22—C21	−177.8 (3)
C14—C10—C11—C12	176.8 (4)	C24—O10—C23—O9	0.9 (5)
C1—C2—C10'—C11'	125.0 (18)	C24—O10—C23—C7	−178.3 (3)
C3—C2—C10'—C11'	−91 (2)	C8—C7—C23—O9	153.5 (4)
C10—C2—C10'—C11'	10.6 (14)	C6—C7—C23—O9	−26.5 (5)
C1—C2—C10'—C12	−51 (2)	C8—C7—C23—O10	−27.4 (4)
C3—C2—C10'—C12	93 (2)	C6—C7—C23—O10	152.6 (3)

### Hydrogen-bond geometry (Å, °)

**Table:**

*D*—H···*A*	*D*—H	H···*A*	*D*···*A*	*D*—H···*A*
O3—H3O···O8	0.99 (6)	1.65 (6)	2.541 (4)	149 (5)
C13—H13C···O3^i^	0.96	2.64	3.467 (5)	145
C15—H15C···O2^ii^	0.96	2.53	3.342 (5)	142
C22—H22A···O8^iii^	0.93	2.49	3.316 (5)	149
C24—H24A···O5^iv^	0.96	2.67	3.392 (5)	133

Symmetry codes:  (i) *x*+1/2, −*y*+1/2, −*z*+1; (ii) *x*−1/2, −*y*+3/2, −*z*+1; (iii) −*x*+2, *y*+1/2, −*z*+1/2; (iv) *x*+1/2, −*y*+3/2, −*z*+1.
